# Picornaviruses and Apoptosis: Subversion of Cell Death

**DOI:** 10.1128/mBio.01009-17

**Published:** 2017-09-19

**Authors:** Sarah N. Croft, Erin J. Walker, Reena Ghildyal

**Affiliations:** Respiratory Virology Group, Health Research Institute, Faculty of Education, Science, Technology and Mathematics, University of Canberra, Canberra, Australia; Johns Hopkins Bloomberg School of Public Health; Harvard Medical School

**Keywords:** apoptosis, innate immunity, picornavirus, proteases, virus-host interactions

## Abstract

Infected cells can undergo apoptosis as a protective response to viral infection, thereby limiting viral infection. As viruses require a viable cell for replication, the death of the cell limits cellular functions that are required for virus replication and propagation. Picornaviruses are single-stranded RNA viruses that modify the host cell apoptotic response, probably in order to promote viral replication, largely as a function of the viral proteases 2A, 3C, and 3CD. These proteases are essential for viral polyprotein processing and also cleave cellular proteins. *Picornavirus* proteases cleave proapoptotic adaptor proteins, resulting in downregulation of apoptosis. *Picornavirus* proteases also cleave nucleoporins, disrupting the orchestrated manner in which signaling pathways use active nucleocytoplasmic trafficking, including those involved in apoptosis. In addition to viral proteases, the transmembrane 2B protein alters intracellular ion signaling, which may also modulate apoptosis. Overall, picornaviruses, via the action of virally encoded proteins, exercise intricate control over and subvert cell death pathways, specifically apoptosis, thereby allowing viral replication to continue.

## INTRODUCTION

Picornaviruses can selectively alter cellular pathways in order to promote viral replication, mostly through the action of viral proteases, including modulation of proapoptotic factors or processes. Control of cell death processes is vital to viral replication, which requires a viable cell; however, release of the virus can be inhibited through apoptosis inhibitors (1), and thus, a precise control over pro- and antiapoptotic processes is essential to *Picornavirus* replication. As such, control or modulation of apoptotic processes is favorable to picornaviruses, and this minireview will specifically focus on the modifications of cellular processes by picornaviruses that lead to downregulation of apoptosis. The direct cleavage of caspases (2), disruption of nuclear-cytoplasmic trafficking (3, 4), relocalization of proapoptotic proteins (5, 6), and cleavage of essential apoptotic adaptor proteins (7, 8) have all been shown to occur as a result of action of *Picornavirus* protease activity and together suggest mechanisms by which picornaviruses can alter host cell apoptotic death pathways.

## PICORNAVIRUSES

Picornaviruses cause a wide variety of diseases in humans. Within the respiratory system, human rhinovirus (HRV) causes common colds and asthma (9), within the nervous system, poliovirus (PV) causes poliomyelitis (10), and within the liver, hepatitis A virus (HAV) causes hepatitis (11). Picornaviruses are small, nonenveloped viruses that contain a single strand of positive-sense RNA (ssRNA) (12). The *Picornavirus* replication cycle is initiated by attachment of the virus to the host cell receptor, followed by internalization and uncoating of the virus genome (13). The single open reading frame of the genome is translated into a large polyprotein that undergoes posttranslational processing (*cis* and *trans* cleavage) by the virally encoded proteases to yield mature, structural or nonstructural proteins (14). The mature proteins then participate in transcription and replication of the RNA genome and assembly and release of the virus. The viral proteases not only participate in the maturation of the viral proteins but also act against cellular factors, resulting in host cell shutoff and increased virus replication. Cell death is an important cellular mechanism to limit viral spread, and viruses, including picornaviruses, have evolved to inhibit cell death. In this minireview, we aim to review and discuss current literature on *Picornavirus* modulation of cell death pathways with a view to integrating diverse studies to form a rational informed model of *Picornavirus* disruption of apoptotic pathways.

## APOPTOSIS

Cell death can occur as a result of many stimuli with the type of stimulus determining the type of cell death. Apoptosis is a process of selective, controlled cell death (15–17), which utilizes a cascade of signaling proteins after the activation of a death signal. The induction of apoptosis, discussed below in the context of viral infection, can occur extrinsically, in response to nonself or external stimuli, or intrinsically, in response to inherent, cellular abnormalities (17). The extrinsic and intrinsic apoptosis pathways converge to cleave and activate procaspase-3 to derive caspase 3, which is termed an executioner caspase. Caspase 3 subsequently activates or inactivates substrates to facilitate the morphological changes that are associated with apoptotic cell death, such as chromatin condensation, cell shrinkage, nuclear and plasma membrane blebbing (15, 16, 18). The removal of the resulting apoptotic bodies occurs through phagocytic engulfment ensuring that the process avoids an inflammatory response (15–17). Apoptotic bodies can provide a means for viruses to evade immune responses (19); however, the induction of apoptosis has been shown to reduce viral titer (20). Studies have shown that it is not the complete inhibition of apoptosis that is beneficial to viral replication, but timely control that results in apoptotic delay, then induction (2, 21).

**FIG 1:**
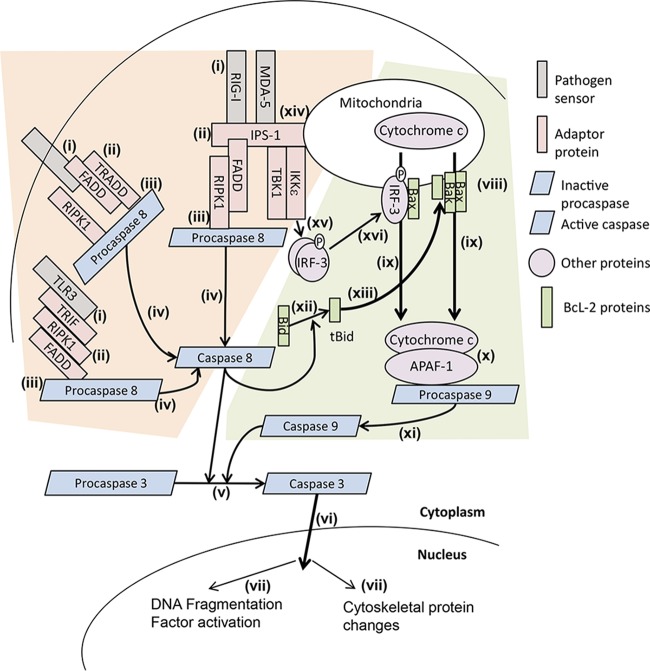
Antiviral apoptotic pathways. Induction of the extrinsic apoptotic pathway (pink shaded area) is initiated by the binding of a picornavirus-associated molecular pattern to a pathogen receptor (i), e.g., dsRNA to TLR3, MDA-5, or RIG-I. Subsequently, adaptor proteins TRIF, FADD, RIPK1, and TRADD are recruited directly to the receptor or via IPS-1 in the case of RIG-I and MDA-5 (ii), forming the death receptor complexes. Procaspase-8 is recruited to the death receptor complexes (iii) and undergoes autocatalysis (iv) to active caspase 8. Caspase 8 then cleaves and activates caspase 3 (v), which translocates to the nucleus (vi) and activates proteins to execute apoptotic morphological changes (vii). The cleavage of Bid by caspase 8 (xii) produces truncated Bid (tBid), which induces Bax and Bak (xiii) to insert into the outer mitochondrial membrane, allowing cytochrome *c* to translocate into the cytoplasm (ix). APAF-1 binds cytochrome *c* (x), and procaspase-9 is recruited to the complex, undergoing autocatalysis to caspase 9 (xi). Caspase 9 cleaves procaspase-3 to caspase 3 (v), which translocates to the nucleus (vi) and activates proteins to execute apoptotic morphological changes (vii). TBK1 and IKKε can also be recruited to IPS-1 (xv), and once recruited, phosphorylate IRF-3. Phospho-IRF-3 translocates to the outer mitochondrial membrane (xvi), complexing with Bax to form a pore (viii), releasing cytochrome *c* into the cytoplasm (ix) and inducing a procaspase-9-dependent apoptotic signaling cascade. Abbreviations: TLR3, Toll-like receptor 3; TRIF, TIR domain-containing adaptor-inducing beta interferon; RIPK1, receptor-interacting protein kinase 1; FADD, Fas-associated protein with death domain; TRADD, tumor necrosis factor receptor type 1-associated death domain protein; RIG-I, retinoic acid-inducible gene 1; MDA-5, melanoma differentiation-associated protein 5; IPS-1, interferon-β promoter stimulator 1; TBK1, TANK-binding kinase 1; IKKε, inhibitor of nuclear factor κB kinase epsilon; IRF-3, interferon regulatory factor 3; Bak, Bcl-2 homologous antagonist/killer; Bax, Bcl-2-like protein 4; Bid, BH3-interacting-domain death agonist; tBid, truncated Bid; APAF-1, apoptotic protease activating factor 1.

Apoptotic death in response to viral infection can commonly proceed via the extrinsic pathway in response to external stimuli or via the intrinsic pathway in response to internal stress signals (Fig. 1). Upon the recognition of foreign molecular patterns by death signaling proteins (e.g., Fas [22]) [Fig. 1(i)], adaptor proteins are recruited, and death signaling complexes form (22, 23) at the mitochondria or in the cytoplasm. The exact composition of death signaling complexes will vary [Fig. 1(ii)]; however, activation results in the recruitment of procaspase-8 [Fig. 1(iii)], which then undergoes autocatalysis to form active caspase 8 [Fig. 1(iv)] (22). Caspase 8 cleaves procaspase-3 into the active caspase 3 [Fig. 1(v)] (24) that causes subsequent characteristic apoptotic changes (24–26) [Fig. 1(vi) and (vii)].

## TOLL-LIKE RECEPTORS IN APOPTOSIS

Toll-like receptors (TLRs) are specialized, cellular sensors that recognize pathogen-associated molecular patterns, with Toll-like receptor 3 (TLR-3) recognizing cytoplasmic double-stranded RNA (dsRNA), a picornaviral replication intermediate (23, 27) (Fig. 1). The binding of dsRNA to TLR-3 induces the formation of a TLR-3 homodimer, allowing exposure of the Toll–IL-1 receptor (TIR) domain (28). TIR domain-containing adaptor-inducing beta interferon (TRIF) binds to TLR-3. TRIF is essential for downstream nuclear factor κB (NF-κB), interferon regulatory factor 3 (IRF-3), and beta interferon (IFN-β) activation (29). The presence of a dominant-negative form of TRIF abolishes NF-κB signaling from TLR-2, -3, -4, and -7 (29), suggesting that it is crucial for a large portion of TLR signaling. Overexpression of TRIF induces apoptosis (29), dependent on its C-terminal domain, between amino acids 532 to 712 (29). TRIF-induced apoptosis relies on functional FADD (Fas-associated protein with death domain) and caspase 8, linking the sensing of dsRNA to apoptosis activation (29, 30) [Fig. 1(i) to (iv)], although other studies have reported that FADD depletion does not prevent TLR-3-induced apoptosis (28). Also essential to this pathway is RIPK1 (28), which associates with the C-terminal region of TRIF (30) as well as FADD and caspase 8 (29). The interaction between TRIF and RIPK1 occurs within homologous RIP homotypic interaction motif (RHIM) domains in both proteins, being mapped to the C-terminal region of TRIF (30) (see Fig. 4A). Mutation in the RHIM domain in TRIF abrogated TRIF-induced apoptosis, indicating that TLR-3-dependent apoptosis proceeds along a TRIF-RIPK1-FADD-caspase 8 axis (29, 30). RIPK1 can commit the cell to multiple cell death pathways; however, the RIPK1-induced cell death following TLR-3 activation was confirmed to be apoptotic and not necrotic (28).

## VIRAL RNA SENSORS IN APOPTOSIS

The activation of either RIG-I (retinoic acid-inducible gene 1) or MDA-5 (melanoma differentiation-associated protein 5), cytosolic 5′-triphosphate RNA, or long dsRNA helicase receptors results in each receptor associating via homologous caspase recruitment domains (CARDs) to a mitochondrion-bound adaptor protein, IFN-β promoter stimulator-1 (IPS-1), from which multiple biochemical pathways can be induced (Fig. 1). Activated IPS-1 recruits IKKε (inhibitor of nuclear factor κB kinase epsilon) and TBK1 [Fig. 1(xv)], which results in the subsequent phosphorylation and dimerization of IRF-3 (31). IRF-3 homodimers can then translocate either to the nucleus, where they induce the expression of IFN-β or complexes with Bax (32) and translocate to the mitochondrial membrane [Fig. 1(xvi)], leading to the formation of a pore and induction of apoptosis. IPS-1-dependent IRF-3 activation has been shown to upregulate the expression of proapoptotic Bcl-2 proteins (discussed later within this minireview) (33), activate proapoptotic BH3 domain-containing proteins (34), and display a mitochondrial localization-dependent ability, leading to the activation of caspase 9 [Fig. 1(x) and (xi)] (35).

Additionally, IPS-1 activation can result in the formation of a complex that contains IPS-1, FADD, caspase 8, and RIPK1 (36) [Fig. 1(ii)]. The downstream effect of this complex relies on caspase 8-dependent cleavage of RIPK1 (36) at aspartate 324 which also acts to negatively regulate the activation of IRF-3 (36). The association of caspase 8 and RIPK1 to the IPS-1/RIG-I/MDA-5 complex induces activation of the apoptotic pathway (37).

Recent work has shown that IPS-1-induced apoptosis proceeds along an IPS-1/RIPK1/caspase 8 axis, independent of any IFN response or intrinsic apoptosis pathway (36, 38). Furthermore, IPS-1 and caspase 8 have been shown to be essential in anoikis, cell detachment due to apoptosis (39) through the adaptor protein DAP3, which is also essential for the recruitment of FADD to other apoptosis-inducing proteins (40); however, further research is needed to confirm the association of DAP3 in dsRNA/IPS-1-induced apoptosis.

Protein kinase RNA-activated (PKR) is a dsRNA sensor important for host responses in *Picornavirus* infection. PKR exists as a latent single molecule; however, the binding of dsRNA to two PKR molecules increases their proximity, thus inducing autophosphorylation and activation (41). The active kinase can activate p53 and NF-κB and has been shown to phosphorylate eukaryotic initiation factor 2a (eIF2a), inactivating it. The inactive eIF2a then acts as a competitive inhibitor of eIF2B, resulting in shutdown of protein synthesis (41, 42). The phosphorylated form of eIF2a has been shown to induce apoptosis through translational control, rather than complete translational shutdown (43, 44). Notably, eIF2a induces the downregulation of translation of antiapoptotic genes and promotes the expression of proapoptotic genes such as Fas, FasL (extrinsic apoptosis pathway), and p53 (intrinsic apoptosis pathway) as reviewed by Gil and Esteban (45). However, the activated apoptotic pathway induced after PKR activation can also proceed in a FADD- and caspase 8-dependent manner in response to viral infection (46) with a role for IPS-1 in the induction of PKR-induced apoptosis (47). Thus, PKR is hypothesized to, like RIG-I and MDA-5, bind and require IPS-1 for downstream signaling to caspase 8, which involves the formation of a complex with FADD, caspase 8, and the caspase 8-generated cleaved RIPK1 fragment, to promote apoptosis.

## INTRINSIC APOPTOSIS

An apoptotic death signal can also be triggered in response to disruption to an internal cellular balance or process such as calcium ion balance disruption (discussed further in this minireview), DNA mutations, or mitochondrial dysfunction [Fig. 1(viii)]. Regardless of stimuli, critical to the intrinsic apoptosis pathway is loss in mitochondrial outer membrane permeability via membrane potential changes or the formation of a pore complex (48). The formation of the pore complex is dependent on the balance between pro- and antiapoptotic Bcl-2 family member proteins as reviewed in reference 49. The antiapoptotic Bcl-2 proteins (for example, Bcl-2 and Bcl-XL) interact with proapoptotic Bcl-2 proteins and act as negative regulators of apoptosis. Two classes of proapoptotic Bcl-2 proteins have been identified: those that act as initiator proteins (for example, Bim, truncated Bid [tBid], and Bad) and those that form the pore within the outer mitochondrial membrane (for example, Bak and Bax). The oligomerization of Bcl-2 proteins on the mitochondrial membrane forms a pore and allows proapoptotic factors into the cytoplasm. Cytochrome *c* is released within the cytoplasm (Fig. 1(ix)) where it complexes with apoptotic peptidase-activating factor 1 (APAF-1) [Fig. 1(x)] and recruits procaspase-9, inducing procaspase-9 autocatalysis and activation [Fig. 1(xi)], which in turn activates caspase 3 [Fig. 1(v)] (50).

## EXTRINSIC AND INTRINSIC APOPTOSIS CROSS TALK

Importantly, there is interplay between the intrinsic and extrinsic pathways, with caspase 8 cleaving a Bcl-2 protein, Bid [Fig. 1(xii)]. Truncated Bid (tBid) then activates other Bcl-2 proteins (Bax and Bak) (51) [Fig. 1(xiii)], resulting in the formation of a pore on the outer mitochondrial membrane [Fig. 1(viii)] and inducing the intrinsic apoptosis pathway (51, 52). Furthermore, mitochondrion-anchored death signaling adaptor proteins have been implicated in response to *Picornavirus* infection that, when activated, can lead to caspase 8 activation {extrinsic apoptosis [Fig. 1(iii) and (iv), pink shaded area]} or the formation of a mitochondrial pore {intrinsic apoptosis [Fig. 1(xv) and (xvi), green shaded area} (34, 36).

## DISRUPTION OF APOPTOTIC PATHWAYS BY PICORNAVIRUSES

### Caspases.

**FIG 2:**
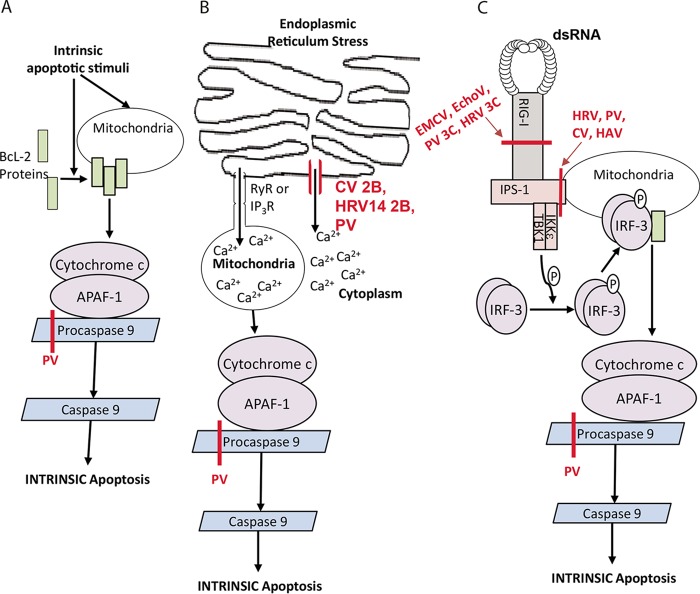
Effects of picornavirus infections on intrinsic apoptosis pathways. (A) Caspase 9-dependent intrinsic apoptosis is initiated by the release of mitochondrial cytochrome *c* into the cytoplasm as described in the legend to Fig. 1. Procaspase-9 is cleaved in PV infection (indicated in red), resulting in a lack of function and inability to activate caspase 3, thus inhibiting intrinsic apoptosis. (B) The abrupt release of Ca^2+^ from the endoplasmic reticulum (ER) is a protective response following ER stress. The released Ca^2+^ is taken up into the mitochondrial matrix via RyR and IP_3_R channels. Increase of Ca^2+^ above a threshold level in the mitochondrial matrix induces an increase in mitochondrial membrane permeability, allowing the cytoplasmic release of cytochrome *c*, inducing intrinsic apoptosis. Picornaviral 2B protein inserts into the ER, forming an ion channel (indicated in red), allowing Ca^2+^ to leak into the cytoplasm, and decreasing the amount of Ca^2+^ available to translocate to the mitochondria, thus inhibiting intrinsic apoptosis. (C) RIG-I and MDA-5, when activated, associate with IPS-1 and recruit TBK1 and IKKε into a death receptor complex at the mitochondria. TBK1 and IKKε act by phosphorylating IRF-3 dimers which then induce a pore in the mitochondrial membrane, allowing release of cytochrome *c* into the cytoplasm, inducing intrinsic apoptosis. RIG-I, MDA-5, and IPS-1 are cleaved during infection (indicated in red) by several picornaviruses, inhibiting intrinsic apoptosis. P, phosphate; EchoV, echovirus.

An early study by Belov et al*.* in 2003 showed that cells infected with poliovirus (PV) did not display apoptotic morphological changes (notably, DNA degradation and caspase 3 cleavage) (2). In contrast, cells that were subject to PV infection and viral (RNA replication) inhibitor guanidine hydrochloride (G-HCl) treatment underwent apoptosis as measured by characteristic DNA degradation (2). Without caspase 3, cells (in response to PV plus G-HCl) did not undergo apoptosis, implicating caspase 3 as the executioner of PV-induced apoptosis. Together these results suggest that apoptosis is induced in response to PV infection but active PV infection is able to inhibit apoptosis progression. PV infection resulted in an efflux of cytochrome *c* from the mitochondria into the cytoplasm (2), which should induce apoptosis (53). PV infection also resulted in the aberrant processing of procaspase-9 that probably contributes to the suppression of apoptosis (2) (Fig. 2A). Although it is not known whether a viral protease is responsible for the aberrant processing of procaspase-9 and there is no further research on this topic, this is a clear example of a picornavirus (either directly or indirectly) targeting caspase activity and altering apoptotic cell death pathways.

### Disruption of calcium ion signaling.

There are multiple pathways that link the endoplasmic reticulum (ER), which is a calcium ion (Ca^2+^) storage site (54), to the induction of apoptosis. One mechanism of apoptosis induction is the sudden release of Ca^2+^ from the ER lumen into the cytoplasm of a cell (Fig. 2B) (55). The abrupt release of Ca^2+^ from the ER acts as a signaling mechanism following ER stress in order to protect the cell or organism from detrimental changes in Ca^2+^ or the misfolding or accumulation of proteins within the ER (54). The released Ca^2+^ is taken up into the mitochondrial matrix; this translocation can occur through electrophoretic mechanisms but predominantly occurs through microdomains (points of contact between the ER and the mitochondria) (54, 55) (Fig. 2B). Ca^2+^ release from the ER is dependent on the Ca^2+^ efflux through inositol 1,4,5-triphosphate receptor (IP_3_R) and ryanodine receptor (RyR) channels (56). Increase of Ca^2+^ concentration above threshold within the mitochondrial matrix then induces an increase in mitochondrial membrane permeability through the voltage-dependent anion channel (VDAC) in the outer mitochondrial membrane within the permeability transition pore complex (PTPC) (54, 55). This increase of permeability allows the cytoplasmic movement of mitochondrial proapoptotic proteins such as cytochrome *c* (Fig. 2B), inducing intrinsic apoptosis within the cytoplasm when complexed with APAF-1 (55). However, reports also show that this apoptosis pathway can proceed in an APAF-1-independent manner (54), indicating the presence of another Ca^2+^-dependent apoptosis pathway.

Picornavirus infection results in an increase in cytoplasmic Ca^2+^ concentrations dependent on viral gene expression (56, 57), probably through 2B protein activity (58) (Fig. 2B). Coxsackievirus B 3 (CVB3) 2B protein consists of two hydrophobic regions and inserts into Golgi complex and ER membranes as an integral membrane pore (Fig. 2B) (58, 59). 2B expression leads to downregulation of Ca^2+^ flux between intracellular stores and the mitochondria, proposing a mechanism by which the protein could suppress intrinsic, Ca^2+^-induced apoptosis (Fig. 2B) (56, 59). The Ca^2+^ released via 2B pores is not taken up by the mitochondria, as would normally occur in release via IP_3_R and RyR (56). The importance of 2B protein is exemplified by the fact that *Picornavirus* 2B proteins are highly conserved (58). The CV, PV, and human rhinovirus (HRV) 2B proteins all contain similar, functionally important, conserved hydrophobic domains (58). Paralleling this, subcellular localization studies showed that PV and HRV14 2B localize to the Golgi complex and disrupt the ER Ca^2+^ stores (58). The disruption of subcellular Ca^2+^ concentrations could be a mechanism by which picornaviruses are able to suppress the intrinsic apoptotic response, by subverting Ca^2+^ signaling to the mitochondria.

### dsRNA sensors.

RIG-I and MDA-5 are implicated in host responses to *Picornavirus* infection (27). The activation of RIG-I in *Picornaviridae* infection has been debated with Wang et al. (99) indicating that RIG-I is not activated upon HRV infection, but Slater et al. (27) showing that RIG-I and MDA-5 activation occurs later in infection, coordinating with TLR-3 activation. The mechanism by which endosomally located TLR-3 recognizes cytoplasmic HRV dsRNA is unknown; however, it has been proposed that RNA secondary structures formed by the single strand within the endosome may be recognized by TLR-3 (27).

**FIG 3:**
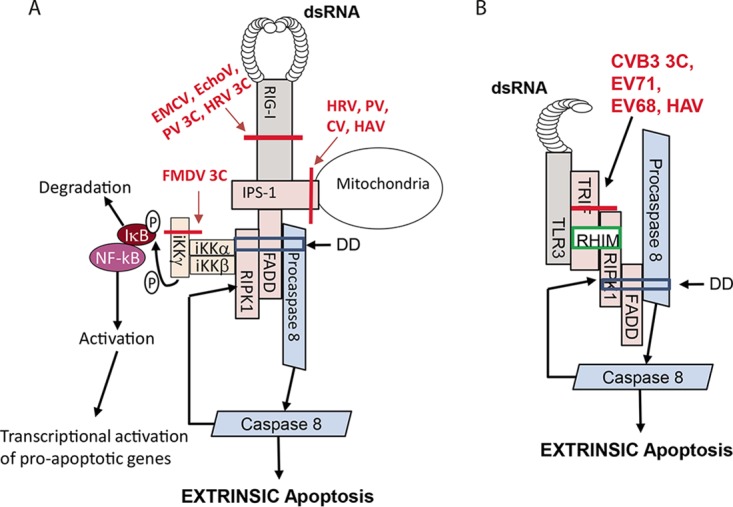
Effects of picornavirus infections on extrinsic apoptosis pathways. (A) Sensing of dsRNA by RIG-I/MDA-5 leads to the formation of a death receptor complex at the mitochondria through the adaptor protein IPS-1. Subsequent ubiquination of RIPK1 is recognized by the IKKγ (Nemo) subunit of the IKKα-IKKβ-IKKγ complex, leading to phosphorylation and degradation of IκB and release of NF-κB that translocates into the nucleus to upregulate expression of proapoptotic genes. Cleavage of RIG-I and IPS-1 by various picornaviruses (indicated in red) disrupts extrinsic apoptosis signaling. Foot-and-mouth disease virus (FMDV) infection results in cleavage of IKKγ, abrogating NF-kB activation and attenuating transcriptional activation of proapoptotic factors. (B) Sensing of dsRNA by TLR3 and subsequent recruitment of TRIF initiates the formation of a death receptor complex, leading to induction of extrinsic apoptosis as described in the legend to Fig. 1. TRIF is cleaved in infection by multiple picornaviruses (indicated in red) resulting in disruption of the death receptor complex and inhibition of extrinsic apoptosis. P, phosphate; DD, death domain; RHIM, RIP homotypic interaction motif.

The importance of picornaviruses attenuating this pathway is highlighted by the multiple components that are cleaved by viral proteases (Fig. 2C and 3A and Table 1). The RNA sensors are cleaved by multiple picornaviruses. Barral et al. (60) have shown that RIG-I is cleaved during PV, major and minor group HRV, encephalomyocarditis virus (EMCV), and echovirus infection (61). Furthermore, exogenous addition via transfection or *in vitro* addition of the PV 3C protease leads to degradation of RIG-I similar to that seen in infection, indicating that it is the most likely cause of cleavage in *Picornavirus* infection (60). Multiple viruses have been shown to cleave IPS-1, thus disrupting virus-induced downstream signaling. Notably, HRV1a, PV, coxsackievirus (8, 62), and hepatitis A virus (HAV) (63) proteases have been shown to cleave IPS-1, all disrupting downstream functions (Fig. 2C). Hepatitis C virus (HCV) (a flavivirus), protease NS3/4A has been shown to cleave IPS-1 near the N-terminal transmembrane domain (64) which disrupts mitochondrial localization of IPS-1 (64), essential for downstream signaling (65, 66). While limited data are available, IPS-1 is cleaved in HAV infection in a similar manner and releases IPS-1 from the mitochondria (63), possibly disrupting intrinsic apoptosis.

**TABLE 1:** Proapoptotic factors cleaved in picornavirus infection

Protein	Virus(es)	Protease(s)	Reference
RIG-I	PV	3C	60
	HRV16, -1a		
	EMCV		
	Echovirus		
	EMCV	3C	61

IPS-1	HRV1a	3ABC, 2A	62
	PV	3C, 3ABC	
	CVB3	2A	
	HAV	3ABC	63
	CVB3	3C	8

IKKγ	FMDV	3C	7

TRIF	CVB3	3C	8
	EV71	3C	67
	EV68	3C	68
	HAV	3CD	69

Sam68	FMDV	3C	95
Procaspase-9	PV		2

Cleavage of IPS-1, releasing the death receptor complex from its mitochondrial anchor would also be expected to result in inhibition of extrinsic apoptosis (Fig. 3A). In addition, picornaviruses target the helicase activation-dependent proinflammatory pathways. Ubiquination of RIPK1 at lysine 377 acts as a scaffold which is recognized by the IKKγ (Nemo) subunit of the IKKα-IKKβ-IKKγ complex (Fig. 3A), activating the latter which allows the subsequent phosphorylation and degradation of iKB (31), releasing NF-κB which translocates into the nucleus to upregulate the expression of proinflammatory genes. Foot-and-mouth disease virus (FMDV) 3C protease cleaves IKKγ, effectively disrupting the activation of NF-κB (Fig. 3A).

### TLR pathways.

TRIF is cleaved at two sites within its N-terminal and C-terminal regions in cells infected with CVB3 (8) (Fig. 3B). Ectopic expression of the CVB3 3C protease confirmed it as the enzyme responsible for cleavage (8). The same report also noted that the virus-induced truncated form of TRIF was unable to mount IFN or apoptotic responses, likely due to the cleavage at the C-terminal region of TRIF (8). The picornaviral cleavage of TRIF has also been shown in EV71 (67), EV68 (68), and HAV (69) infection, suggesting that it may be common to *Picornaviridae*. Additionally, it was confirmed by multiple sources to be the result of 3C protease (8, 67, 68) or its precursor 3CD protease (69) activity. Whether the viral alteration of TRIF, downregulation of IFN responses reported in EV71 and EV68 infection (67, 68), and impaired IRF-3 responses in HAV infection (69) lead to disruption of apoptosis was not tested. The cleavage of TRIF by HAV occurs at position 554 (69), which is within the proapoptotic C-terminal domain of TRIF (29); thus, the cleavage of adaptor proteins within this pathway may be a mechanism of modulating the host apoptotic response.

### Nuclear cytoplasmic trafficking and apoptosis.

The genomic content in eukaryotes is separated from the rest of the cell by the double lipid bilayer of the nuclear envelope (NE). The NE is interrupted by pores, the only avenue for bidirectional transport of molecules across the NE (70). The nuclear pore complex (NPC), is comprised of approximately 500 to 1,000 nucleoporins (Nups), of which there are approximately 30 types (reviewed in references 70 and 71). NPCs contain a hollow central core, a nuclear basket, a lumenal ring, and cytoplasmic filaments. Nups lining the central core have numerous phenylalanine and glycine repeats (FG Nups) and mediate nucleocytoplasmic transport via interaction with nuclear transporters termed importins (IMPs) or exportins (EXPs) (71). The nuclear import cycle is mediated by direct binding of IMPβ to cargo molecules through their nuclear localization signals (NLSs) or indirect binding via an adaptor protein (IMPα). Cytoplasmic filaments provide the initial docking site for the import complex which moves through the NPC facilitated by the FG Nups. In the nucleus, RanGTP binds to the IMPβ complex which induces cargo displacement, and IMPβ-RanGTP is transported back into the cytoplasm. Nuclear export is similar to nuclear import in the opposite direction. The best-characterized EXP is CRM1 that recognizes proteins with a leucine-rich nuclear export signal (NES) and in complex with RanGTP, translocates the complex to the cytoplasm through the NPC. In the cytoplasm, RanGTP is hydrolyzed to cause dissociation of the CRM1-RanGTP-cargo complex. The unbound CRM1 and RanGDP are free to recycle back into the nucleus to repeat the cycle.

Procaspase-3 is located in cytoplasmic fractions of nonapoptotic cells and the nuclear fraction of apoptotic cells (72), indicating its nuclear translocation in response to apoptotic stimuli, correlating with the nuclear location of caspase 3 substrates (73). Caspase 3 harbors a NES that keeps it in the cytoplasm in its procaspase form. Activation of caspase 3 followed by a specific substrate recognition leads to masking/abrogation of the NES activity, resulting in nuclear localization of caspase 3 (73–75).

Protein kinase Cδ (PKCδ) is a proapoptotic protein that accumulates in the nucleus during apoptosis. Full-length PKCδ is found in all cells, whereas the catalytic fragment of PKCδ, generated by caspase cleavage, is present only in cells undergoing apoptosis. PKCδ normally shuttles between the nucleus and the cytoplasm. In cells undergoing apoptosis, it is cleaved by caspase 3 in the nucleus, disrupting its NES and resulting in nuclear accumulation (76, 77).

### Disruption of nucleocytoplasmic trafficking.

Many picornaviruses cleave or alter proteins involved in nucleocytoplasmic transport (as reviewed in reference 78), thereby disrupting host processes and promoting viral replication. The extent that viral modulation of NPCs causes disruption of apoptosis pathways has not been investigated extensively; however, the orchestrated nucleocytoplasmic transport of pro- and antiapoptotic factors has been studied and reviewed (79–81).

Cells infected with PV have mislocalization of select endogenous NLS-containing proteins and a green fluorescent protein (GFP)-tagged reporter protein containing an NLS to the cytoplasm due to the inability of import complexes to dock to the NPC (82). Coinciding with the decrease in nucleocytoplasmic transport, key FG Nups (71, 83, 84), Nup153, and Nup62 were degraded in PV infection in a manner dissimilar to that seen in apoptosis (82). Studies have since shown cleavage of Nup153, -98, and -62 in HRV infection (3, 4), with HRV 2A protease likely responsible for Nup62 and Nup98 cleavage (85, 86) and HRV 3C protease likely responsible for Nup153 cleavage (3). Additionally, HRV 3C protease increased the permeability of the NE to large molecules (>100 kDa) in a perforated cell system (87). Together, these results indicate that cytoplasmic accumulation of nuclear or NLS-containing proteins and nuclear accumulation of molecules that cannot normally access the nucleus are, at least partially, due to the cleavage of Nups by picornaviral proteases.

Notably, an increase in nuclear permeability is also seen in infection with cardioviruses, specifically encephalomyocarditis virus (EMCV) (88) and Theiler’s murine encephalomyelitis virus (TMEV) (89). Cardioviruses lack a protein homologous to the enterovirus (e.g., PV and HRV) 2A protease, and instead the NE alterations are dependent on the viral leader (L) protein (88, 89). Albeit by different mechanisms, the increased permeability of the NPC appears to be conserved among several *Picornavirus* genera, implying that this change is an important modification in *Picornavirus* infection.

While the mislocalization of specific proapoptotic proteins as a result of alterations to the NPC by picornaviruses is not fully understood, the orchestrated manner by which these proteins move into the nucleus may be affected by virus-induced changes.

### Picornavirus-induced relocalization of Sam68.

**FIG 4:**
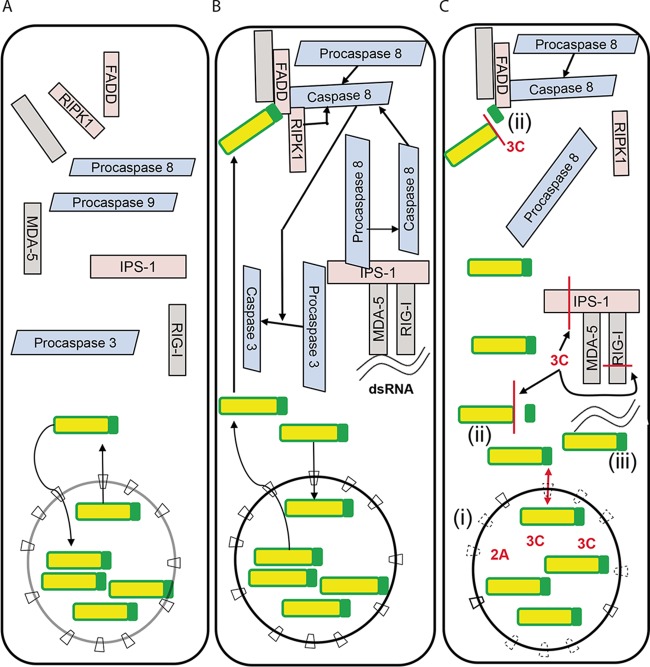
Effects of picornavirus infections on nuclear pore and Sam68. (A) Sam68 (yellow and green) shuttles between the cytoplasm and nucleus, being located predominately in the nucleus in healthy cells, with cytoplasmic cell death proteins in precursor, inactive form. (B) With the detection of dsRNA by RIG-I/MDA-5 or TLR-3, procaspase-8 is recruited to the complex formed, inducing activation of caspase 8 and recruitment of death receptor proteins, e.g., FADD and RIPK1. Death receptor complex signaling is dependent on recruitment of cytoplasmic Sam68. (C) Disruption of the NPC in picornavirus infection (i) (indicated by broken outline of NPCs) allows passive movement of Sam68 between the nucleus and the cytoplasm (as indicated by the red double-headed arrow). The NLS motif of Sam68 (green) is cleaved in picornavirus infection (ii), resulting in reduced recruitment of RIPK1 to the FADD complex, rendering it inactive. Loss of the NLS motif also inhibits the active import of Sam68 into the nucleus (ii), retaining it in the cytoplasm. Last, the viral genomic element, IRES, binds Sam68 within the cytoplasm (iii), allowing viral replication.

Early reports drew a link between apoptosis and Sam68 (*S*rc-*a*ssociated substrate in *m*itosis of *68* kDa), as knockout of Sam68 led to transformation of fibroblasts into a neoplastic phenotype (90). Later research confirmed the role of Sam68 in apoptosis, with overexpression of Sam68 arresting cell cycle progression and inducing apoptosis (91). Sam68 also acts as an adaptor protein for the formation of tumor necrosis factor alpha (TNF-α)- and TLR-3-induced death receptor complexes (Fig. 4A and B) (92, 93). Recruitment of receptor-interacting protein kinase 1 (RIPK1) to the apoptosis-inducing receptor complex appears to be dependent on Sam68. Although caspase 8 is able to bind FADD (Fas-associated adaptor protein with death domain) in the absence of Sam68, the complex is biologically inactive (93).

Sam68 is mislocalized in *Picornavirus* infection (Fig. 4C), specifically in enterovirus 71 (EV71) (6, 94), HRV serotypes 2, 16 (5), and 14 (4), foot-and-mouth-disease virus (FMDV) (95), and PV (96) infection. Initially, disruption of the nuclear accumulation of Sam68 by picornaviruses was attributed to the disruption of the NPC by the viral proteases [Fig. 4C(i)] (4); however, recent research shows that the cytoplasmic redistribution of Sam68 precedes nucleoporin cleavage (95). The latter study also confirmed direct cleavage of Sam68 by FMDV 3C protease [Fig. 4C(ii)], which disrupts the NLS within the Sam68 C-terminal domain (95). The observed mislocalization of Sam68 in picornavirus-infected cells is probably due both to the disruption of NPC and the loss of its NLS; the former may allow Sam68 to diffuse across the NE in both directions, while loss of the NLS would retain it in the cytoplasm. It is also important to note that Sam68 is involved in viral replication within the cytoplasm; during infection, Sam68 interacts with viral elements, the internal ribosome entry site (IRES) [Fig. 4C(iii)] and the viral RNA-dependent RNA polymerase 3Dpol (95–97), and cytoplasmic virus-induced stress granules (97, 98), and sequestration of cytoplasmic Sam68 by viral structures may interfere with recruitment to death signaling complexes.

## CONCLUSION

The *Picornavirus* proteases are not only essential for cleavage of viral polyprotein but they also target cellular factors to shut down cellular processes disruptive to the virus. Ultimately, the viral control of cell death pathways, in particular apoptosis, is essential for optimal replication of picornaviruses. It is both the timely inhibition and then the induction of apoptosis that are thought to allow optimal viral replication, and it is evident that the suppression of proapoptotic factors, as discussed in this minireview, is a viral strategy to shut down innate immune responses. Overall, understanding of the actions of the viral proteases contributes to the understanding of establishment of infection and subsequent pathogenesis, an essential first step toward viable therapeutic strategies.
